# A Routing Protocol Based on Energy and Link Quality for Internet of Things Applications

**DOI:** 10.3390/sl30201942

**Published:** 2013-02-04

**Authors:** Kassio Machado, Denis Rosário, Eduardo Cerqueira, Antonio A. F. Loureiro, Augusto Neto, José Neuman de Souza

**Affiliations:** 1 Federal University of Minas Gerais, Av. Antonio Carlos, 6627, CEP 31270-901, Belo Horizonte,Brazil; E-Mail: loureiro@dcc.ufmg.br; 2 Federal University of Para, Av. Augusto Correa, 01, CEP 66075-110, Belem, Brazil;E-Mails: denis@ufpa.br (D.R.); cerqueira@ufpa.br (E.C.); 3 Institute of Computer Science and Applied Mathematics, University of Bern, Neubrückstrasse 10,3012, Bern, Switzerland; 4 Federal University of Ceara, Campus do Pici, Bloco 942-A, CEP 60020-181, Fortaleza, Brazil;E-Mails: augusto@deti.ufc.br (A.N.); neuman@ufc.br (J.N.)

**Keywords:** energy-efficiency, internet of things, link quality, routing, wireless sensor network

## Abstract

The Internet of Things (IoT) is attracting considerable attention from the universities, industries, citizens and governments for applications, such as healthcare,environmental monitoring and smart buildings. IoT enables network connectivity between smart devices at all times, everywhere, and about everything. In this context, Wireless Sensor Networks (WSNs) play an important role in increasing the ubiquity of networks with smart devices that are low-cost and easy to deploy. However, sensor nodes are restricted in terms of energy, processing and memory. Additionally, low-power radios are very sensitive to noise, interference and multipath distortions. In this context, this article proposes a routing protocol based on Routing by Energy and Link quality (REL) for IoT applications. To increase reliability and energy-efficiency, REL selects routes on the basis of a proposed end-to-end link quality estimator mechanism, residual energy and hop count. Furthermore, REL proposes an event-driven mechanism to provide load balancing and avoid the premature energy depletion of nodes/networks. Performance evaluations were carried out using simulation and testbed experiments to show the impact and benefits of REL in small and large-scale networks. The results show that REL increases the network lifetime and services availability, as well as the quality of service of IoT applications. It also provides an even distribution of scarce network resources and reduces the packet loss rate, compared with the performance of well-known protocols.

## 1. Introduction

The popularization of wireless and sensor technologies, allied to the demand for new Internet of Things (IoT) applications, is creating a new ubiquitous and smart IoT applications era. The IoT [1] is composed of a set of technologies that provide connectivity at all times, everywhere, and about everything. IoT is based on the principle that objects or things interact and cooperate with each other by using wireless links to ensure ubiquitous communications. They might be Radio-Frequency IDentification (RFID) tags, sensor nodes, actuators, or mobile phones, among others. In this context,Wireless Sensor Networks (WSNs) [2,3] play an important role in providing ubiquitous computing that's capable of connecting both real and virtual worlds.

WSNs/IoT applications have a great impact on the quality of life of people and also lead to economic benefits. Thus, IoT/WSNs are attracting considerable attention from universities, industries and governments in assisting the development of new technologies and applications, such as comfortable homes and offices, healthcare, environmental monitoring and smart cities. For example, the ubiquitous systems and wireless sensor technologies offer suitable solution for improving the efficiency of the food supply chain [4,5]. In the case of applications for healthcare, patients can carry medical sensors to monitor key parameters, such as body temperature, blood pressure, ECG (electrocardiogram) and breathing. Furthermore, medical centers will be able to perform advanced remote monitoring to assess patients condition [6]. Regarding real implementations, the Smart Santana project [7] proposes an experimental research facility in a city to support typical applications and services for smart cities [8]. The facility comprises more than 20,000 IoT devices, divided into topologies that have tens or hundreds of nodes, depending on the applications, such as environmental monitoring and smart parking.

In many IoT applications, the sensed data must be sent to the Base Station (BS) for further operations. This should be accomplished through efficient routing protocols that are key components to improve the data transmission, energy-efficiency, and scalability in WSNs. However, the characteristics of WSNs/IoT raise many challenges in designing efficient communication protocols, owing to limited resources and the unreliability of low-power wireless links that typically lack in terms of Quality of Service (QoS)requirements. At the same time, there still remains a need to find a multipath-aware routing protocol that assures data transmission with low delay, latency, loss rate and minimum energy consumption for various IoT applications [9].

In the context of routing protocols, one important criterion used in the route discovery process is the quality estimation of the communication links between nodes. This quality is usually measured as a single value, such as Received Signal Strength Indicator (RSSI) or Link Quality Indicator (LQI) [10]. However, LQI/RSSI only represents a snapshot at a specific point in time for one link between two nodes, and lacks any additional information about remaining energy, hop count and end-to-end. Thus, there is still an urgent need to find a reliable scheme to estimate the end-to-end link quality based on information of different layers (cross-layer) [11], and it is important to enable the nodes that can transmit packets with a high degree of reliability, while increasing the network lifetime and the overall QoS support for IoT applications.

According to Rocha *et al.* [12], as a result of recent technological advances in embedded systems,the processing and memory constraints in WSNs are tending to disappear. However, the problem of energy constraint remains a critical issue. Furthermore, an external energy supply is usually unavailable and the replacement of batteries is not feasible for large-scale networks as expected for IoT systems. Thus, two of the main design objectives of WSN/IoT applications are to reduce energy consumption and prolong the network lifetime.

Another important issue for WSNs/IoT applications is how to mitigate the energy hole or hot-spot problem [13], where the nodes located closest to the BS or in the most used paths tend to use up their energy resources prematurely [14]. This leads to a loss of connectivity between a node near to BS and the packets that are sent but not received at BS, which causes a wasteful of energy and wireless resources. Thus, the route selection scheme must consider the residual energy and end-to-end link quality to avoid the energy holes, while at the same time providing load balance and fair distribution of the scarce network resources.

Given the characteristics outlined above, a routing protocol for WSN/IoT scenarios must also minimize the signaling overhead, which is responsible for increasing the processing and energy consumption in the nodes as well as increasing the packet loss and delay in the network. Additionally,reliable routes must be selected by using a mechanism to estimate the end-to-end link quality, base don cross-layer information, such as network conditions, energy and hop count. However, the current routing protocols for IoT applications do not take into account these key features, and novel solutions must be created.

This article addresses the problems of ensuring reliability, together with energy-efficiency and load balancing in flat-based (homogeneous nodes) WSN/IoT architectures, by proposing an extended version of a routing protocol based on energy and link quality information (REL) [15]. REL aims to overcome the drawbacks that have been discussed earlier and allows the data transmission with low latency,packet loss, and high reliability, as well as a fair distribution of wireless resources, while increasing the network lifetime, for various flat-based IoT applications, such as smart parking, intrusion detection,and monitoring of river flows.

REL proposes an end-to-end route selection scheme based on cross-layer information with a minimum overhead. To achieve energy-efficiency, the nodes send their residual energy to neighboring nodes by means of an on-demand piggyback scheme. Additionally, REL uses an event-driven mechanism to provide load balancing and avoid the energy hole problem.

This article extends our previous work [15] as follows: (*i*) it optimizes the process of route selection by means of end-to-end link quality evaluation and energy information; (*ii*) it employs a new method for smoothing the link quality estimation; and (*iii*) it conducts an in-depth analysis of the proposed solution for large-scale flat-based networks. It presents new simulation and testbed experiments to show the effects and benefits of REL for IoT systems. The results show that, compared with the performance of other well-known routing protocols, REL increases the network lifetime, enhances service availability,and ensures QoS support for IoT applications, while reducing the packet loss rate and signaling overhead.

The remainder of this paper is structured as follows. Section 2 outlines the existing routing protocol sand their drawbacks compared with REL. Section 3 describes the proposed routing by the energy and link quality (REL) protocol for IoT applications. Section 4 shows simulation and testbed experiments. Section 5 summarizes the main contributions and results of this article.

## 2. Related Work

Routing protocols for WSNs can be classified into flat, location-based and hierarchical/cluster categories [16]. The flat structure can be considered a suitable solution for many IoT applications (in network with homogeneous nodes), such as comfortable homes and offices, healthcare, environmental monitoring, and many smart city services. Many applications employed in these scenarios have low tolerance for packet delay and loss.

Routing protocols based on clustering are an alternative to improve QoS and energy consumption fora set of IoT applications [17], such as multimedia-based fire detection [18]. A hierarchical architecture has nodes with different roles or functionalities (heterogeneous nodes, which can be classified feather-head and non-head nodes). They work based on clustering sensor nodes, where the nodes of the cluster communicate with each other (sensor-to-sensor), and mainly with the leader node (cluster-head),responsible for communicating outside the cluster (sensor-to-BS). Some nodes (cluster-head) can have cameras and extra batteries [19]. There are many algorithms for the election of cluster-head, Bacchanalian the key features as follows: residual energy, link quality and location [20]. These algorithms require time for cluster formation, generating additional delay and complexity, which are unsuitable for many IoT applications.

The architecture of routing protocols based on collecting trees is widely used in WSNs. In this approach, sensor nodes share the same specific destination node or BS. Existing solutions, such as CTPNoe [21] and Multi hop LQI [22], dynamically construct a tree of multiple hops for routing messages and data, and, naturally create a traffic pattern of many-to-one. This feature inhibits their general use in IoT due to the dynamic features of their applications, such as smart parking and environmental monitoring. Routing solutions for these scenarios should consider different traffic patterns, for instance one-to-many,many-to-one and many-to-many. In this case, the tree collection protocols represent a solution applicable to a set of IoT applications not addressed in this article.

The different routing protocol architectures offer many techniques to increase the network lifetime and improve the QoS. The use of these architectures should be observed according to the characteristics of the applications, including their goals and QoS requirements. Flat-based approaches represent a suitable solution for many homogeneous IoT scenarios, especially due their low operational complexity and high efficiency. Several attempts have been made to achieve satisfactory results by using flat architecture for routing protocols in WSN/IoT applications.

Most of the works that have been carried out are based on AODV (The implementation of the AODV protocol is available for downloading at http://www.gercom.ufpa.br/downloads/wsn/) (*Ad hoc* On demand Distance Vector) protocol [23], which was originally proposed in RFC 3965. The popularity of AODV is due to its well-defined structure and low complexity. In AODV, on-demand routes can rediscovered, which decrease the overhead, by using pairs of Route Request (RREQ) and Route Reply (RREP) messages. However, the route selection process is only carried out on the basis of the minimal number of hops, which is not suitable for ensuring energy-efficiency and reliable data transmission. This can be explained by the fact that in many cases, a short route, in terms of hops, can be more susceptible to packet loss, due to both noise and interference that affect the link quality. The lack of self-sufficiency results in energy holes and an uneven distribution of scarce network resources. Moreover,AODV only stores one possible route for a given destination node. This means that if a single route fails or is unavailable, a new route must be discovered, which requires more time and increases the delay or failure rate of data delivery.

Among the protocols for WSNs/IoT that evaluate other metrics apart from the number of hops, LABILE (The implementation of the LABILE protocol is available for downloading athttp://www.gercom.ufpa.br/downloads/wsn/) (Link Quality-Based Lexical Routing) [24] can be pointed out. LABILE proposes a routing algorithm based on lexical structures and link quality evaluation. Through the use of LQI, *i.e.*, a metric provided by the physical layer of IEEE 802.15.4 standard [25],LABILE is able to evaluate the link quality. The LABILE proposal evaluates end-to-end link quality, by classifying the possible values of LQI into good or bad. In specific terms, it determines a threshold value for link classification, where the lowest values of LQI (below the threshold) are considered bad, and represent links that are more susceptible to packet loss. During the route discovery process, all the bad links are counted, recorded and reported with the aid of an additional field in RREQ and RREP messages,which is called *Weak Links*. The purpose of LABILE is to select routes with good link qualities. However,this behavior implies that these routes have an exhaustive use, and lead to the premature death of these nodes. This is due to a lack of mechanisms for determining when there is a need to use alternative routes. Thus, LABILE does not take account energy-efficiency into account during the route selection process and load balancing mechanisms as expected for multipath WSN/IoT scenarios.

The EEURP (Energy Efficient Unicast Routing Protocol) [26] proposes a cost function to select routes based on hop count, the average energy consumption in the end-to-end path, and the minimum energy level. EEURP also uses additional fields for RREQ and RREP messages, which report the total amount of residual energy of a path and the lowest energy level along a route. The minimum energy of a path shows if there is a hop with a critical level of energy. The route discovery mechanism takes into account that only the destination node answers the RREQ messages, *i.e.*, the intermediate nodes that have an available route to the destination do not reply by using a RREP message. This approach is required to calculate the energy level of the entire route, although it creates an extra signaling overhead, leading to an additional expenditure of energy and congestion in the WSNs. The main drawback of EEURP is the fact that it does not include a mechanism to estimate the link quality level. EEURP is focused only on the network lifetime and does not consider QoS support for IoT applications. Despite being specified for WSNs, EEURP was evaluated with the aid of the IEEE 802.11 radios, which have a higher transmission power and more bandwidth than typical radios used for WSNs/IoT applications.

A routing and reliable transmission scheme for IoT applications based on percolation theory and small-world concept was proposed in [27]. In the routing process, nodes broadcast probe messages to discover available routes and select them based on a small-world strategy. The proposed routing scheme is able to operate with multiple paths, but it does not provide load balancing techniques to provide a fair and efficient usage of resources in multipath scenarios as expected for IoT applications (and avoid the hot-spot problem). Mechanisms for link evaluation and residual energy are also not included in the route selection.

A routing protocol based on three possible routing techniques is presented in [22]. The routing schemes are the following: simple routing, based on Round-Robin, and weighted-Round Robin. In simple routing models, each network node selects a single node to route all packets to each round. In the Round-Robin routing, each source node has to elect two or more nodes to route each packet using a per-packet load balancing method. This technique considers that the application should allow the arrival of packets out of order in your destination; however, this technique cannot be feasible for many IoT applications. The weighted Round-Robin routing provides load balance mechanism that assigns a weight to each routing node proportionally to metric values. Although the protocol uses residual energy as routing metric, it does not present solutions for the problem of energy hole. Furthermore, the evaluation of the quality of link used in the experiments does not represent the end-to-end link quality during the process of route selection.

Given the requirements, advances and challenges discussed above, it is possible to identify that existing routing schemes lack in providing an integrated QoS, reliability and energy-efficiency onflat-based solutions for WSNs/IoT applications. With this goal in mind, we propose a cross-layer routing mechanism with QoS, multipath support, resilience and energy-aware for WSNs with asymmetric link quality evaluation as expected and required for IoT scenarios.

## 3. A Routing Protocol Based on Energy and Link Quality (REL)

This section outlines a routing protocol based on energy and link quality (REL) (The implementation of REL is available for downloading at http://www.gercom.ufpa.br/downloads/wsn/) for WSNs/IoT applications, such as comfortable homes and offices, healthcare, environmental monitoring and smart cities. REL uses the link quality of wireless links and residual energy during the routing selection process to increase the system's reliability and assure QoS support for IoT applications. Additionally, it includes an event-driven mechanism to provide load balancing and avoid the premature death of nodes/networks.

### 3.1. Link Quality Estimation

Links in WSN communications are typically unreliable, as they often experience fluctuations in quality and weak connectivity. The unreliability of the link is partly due to the use of low-power radios,which have been shown to be very sensitive to noise, interference, and multipath distortion [28]. In this context, the efficiency of the route selection scheme of a routing protocol depends on the accuracy of the LQE (Link Quality Estimator) to increase the protocol reliability [29]. As mentioned earlier, the link quality is usually measured as a single value, such as RSSI or LQI. However, a single LQE value only represents a snapshot at a specific point in time and does not supply any additional information about the remaining energy, hop count and end-to-end link quality.

The route discovery process must rely on the LQE by means of cross-layer information. In this scenario, nodes must be able to perceive the network conditions, estimate the end-to-end link quality, and have some knowledge of the remaining energy of their neighbors node. They also estimate the number of hops required for each possible path, before they can reach the destination node. Following this approach, nodes must dynamically plan and adapt the routing selection process and perform appropriate decisions for assuring QoS and energy-efficiency for IoT applications.

When analyzing a single link, REL relies on LQI, which is a metric provided by the physical layer of the IEEE 802.15.4 standard. LQI ranges from 0 (worst) to 255 (best) and is calculated on the basis of RSSI, SNR (Signal-Noise-Ratio) or a combination of both metrics. Gomez *et al.* [11] show the importance of LQI, and how it can improve the reliability of a single-hop link, where it compares the use of RSSI and LQI. As result, it study found that LQI outperforms RSSI when a correlation is made between the LDR (Link Delivery Ratio) and PER (Packet Error Rate) results [11].

**Figure 1.**
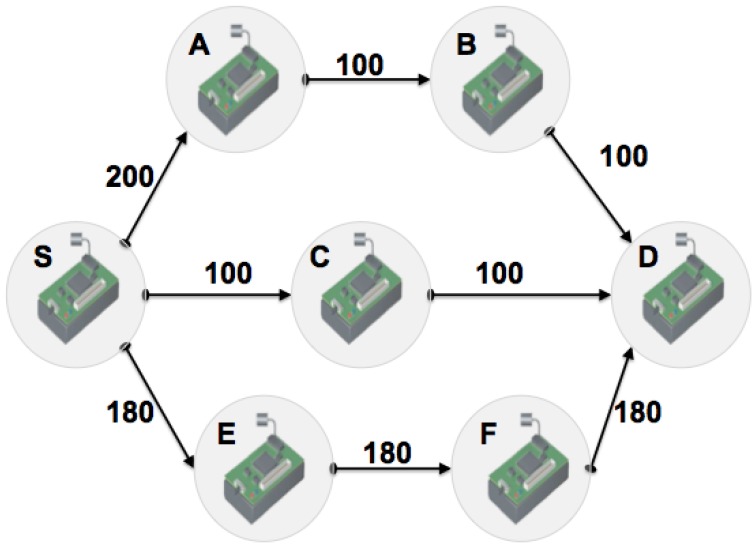
End-to-end link quality estimation.

However, LQI provides a value for the neighboring nodes link quality (not end-to-end). Thus,to increase the reliability of the route discovery process, we need novel techniques to evaluate and collect information about the end-to-end link quality without increasing the signaling overhead. Figure 1 shows the importance of route selection based on cross-layer information and the role of *Weaklinks* in theend-to-end link quality estimation. In Figure 1, the numbers close to the edges represent the LQI value sand nodes S and D are the source and destination nodes, respectively.

REL proposes the use of *WeakLinks*, which is a counter for the worst links along a path. In specific terms, if the LQI for a given link is less than *LQI_th_* (LQI threshold), the link is considered weak and the *WeakLinks* counter is incremented. The *WeakLinks* is incorporated into the RREQ and RREP messages,and then updated at each hop during the route discovery process. Upon receiving a RREQ or RREPmessage, a node updates its LQI value. Following this, it must calculate whether the LQI is lower than *LQI_th_*, and update the *WeakLinks* if necessary.

Let us assume *LQI_th_* = 170, and according to Figure 1 the route selection process that chooses the path with the lowest number of hops, will select node C as the next hop. However, this node has a lower link quality, which will cause packet losses. On the other hand, a route selection that only uses link quality for the next hop, will select node A as the next hop. However, there are bad links along the remaining path, which can also cause packet loss and uneven usage of scarce network resources. Finally, when the *WeakLinks* metric is analyzed, it can be seen that node A has *WeakLinks* = 2, node C has *WeakLinks* = 2 and node E has *WeakLinks* = 0. Thus, when the links are analyzed and selected from the perspective of end-to-end link quality, the most reliable path is through node E. This route is able to provide higher reliability for data delivery.

Most of applications require high Packet Delivery Ratio (PDR), *i.e.*, higher than 80%, to ensure reliable data delivery/QoS support between nodes, as expected for many WSNs/IoT applications, e.g.,healthcare or intrusion detection. Thus, in this work we conduct experiments to find the optimal *LQI_th_*that can provide a PDR higher than 80% (see Section 4.1).

Additionally, REL has an opportunistic behavior, since it uses all the received packets to continuously analyze the LQI value. As result, this solution provides a more accurate measurer means of frequent updates of the link quality for a given route. In contrast with other schemes, our proposal evaluates routes by using the average of the LQI value of a given destination. REL stores *n* values of LQI for each destination, computes the average rather than individual ones. Using the average values of LQI,it is possible to reduce the risk of constant switching between available routes, causing additional delay,and overhead. Thus, when *WeakLinks* is used, REL is less susceptible to extensive link quality variation.

### 3.2. Path Selection and Load Balancing

The limitations of node hardware and the quality variation of wireless links are a great challenge to providing high service availability [30], especially in WSN/IoT applications, where it is necessary to create mechanisms that can identify and mitigate or solve the energy hole problem. Energy holes can be caused by congestion or overuse of a route leading to the premature death of nodes. As discussed before, routing solutions must employ load balancing mechanisms that are able to divert traffic and, thus,increase QoS (less packet loss and delay rate) and reduce energy consumption.

The main approach for load balancing and fault tolerance in WSNs/IoT applications is the use of multiple paths [9] to slit/control traffic along different routes. Through the use of multiple routes, nodes are able to increase reliability for data transmission and throughput by means of bandwidth aggregation,and balanced energy consumption as well.

REL exploits a reactive scheme to find routes on demand, with the aim of reducing the signaling overhead and improving scalability. The route discovery process involves broadcasting RREQ and RREP messages. These messages search for available routes and assist the route selection process by collecting information about residual energy and link quality. Each received RREP represents an available route to the destination node and, according to the REL configuration, it is possible to store n possible routes to a given destination node.

REL integrates/manages the values of three key metrics to find the best available routes as follows:(*i*) quality of wireless links based on *WeakLinks* metric; (*ii*) residual energy; and (*iii*) hop count to avoid long and inefficient paths. Additional fields are required in RREQ and RREP messages to report these three metrics to each possible route.

The path selection process for REL relies on two thresholds to compare the possible routes. The first one is the hop count threshold *HCdiff_max allow_* (Hop Count Maximum Difference), which determines what is the maximum difference of hops to a given route. The second threshold is *E_th_* (Energy Threshold), which is used in two stages: route selection process and load balancing mechanism.

In the case of the load balancing optimization, the *E_th_* corresponds to the monitoring of energy levels observed in each node individually. When the network starts its execution (bootstrap phase), each node has to store its own percentage of residual energy and, after each *t* time units, the node must compare the current energy level (*E*_(_*_t_*_)_) with what has been previously recorded (*E*_(_*_t_*_–1)_). If the difference between*E*_(_*_t_*_)_ and *E*_(_*_t_*_–1)_ is higher than *E_th_*, it indicates an energy event of discharge or involving the need for a charge of the battery.

The difference between energy levels is called *Ind_RADV_* (Index RADV). The *Ind_RADV_* must be compared with the *E_th_*. If the value exceeds the threshold, a RADV (Route Advisor) message must notify nodes near the place where the energy event occurred. The RADV message gives information about the new value for the residual energy and informs the neighboring nodes, where they should re-assess the use of that node in their routes.

RADV notifications can describe a discharge event if there is a critical point of traffic widely used for message routing. It can also present a recharge event if the node has an alternative energy source. The value assigned to *E_th_* should be aware about the overhead generated by RADV notifications. Low values of *E_th_* ensure a uniform energy consumption between the nodes, despite large numbers of notifications. Moreover, high values assigned to *E_th_* cause large differences in the energy consumption in the nodes. As result, this trade off can affect data traffic with delay or the network lifetime.

Algorithm 1 shows the route selection process, where there are three basic rules. The *Shift To Route* function represents a switch between the active route and candidate route. The three rules filter the routes in accordance with the decision structures shown in Lines 5, 11, and 17. These structures evaluate the candidate route (*R_b_*) and categorize it as their energy level, comparing it with the current active route (*R_a_*).

Line 17 shows the use of *E_th_* as a tolerance parameter for the difference that is acceptable if *R_b_* has less energy. After analyzing the energy level, the algorithm calculates the number of hops and evaluates the quality of the links. This evaluation occurs in Lines 7, 12, and 18, where the threshold used is *HCdiff_max___allow_*. According to this algorithm, the next stage of the evaluation needs only be analyzed if *R_a_* is a route that has more hops (Lines 6 and 12).

The rule described in Line 17 is a special case in the selection process, since it analyzes the cases where *R_b_* has less energy than the active route. In this case, *R_b_* has to replace *R_a_* if the energy difference with regard to the threshold *E_th_* and *R_b_* is considerably smaller (Line 18).

In this algorithm, the use of energy and number of hops thresholds is shown as a fine tuning system,able to configure the way the protocol operates in accordance with the purpose and goals of the IoT application. For example, imagine a scenario with *HCdiff_max allow_* = 7. This allows load balancing operations, because there are a larger number of possible alternative routes. However, it causes an increased delay in packet delivery since (long) routes with more hops are used.



**Algorithm 1** Selection Route Algorithm of REL protocol.
1:Let *HCdiff_max allow_* = Hop Count Maximum2:Let *E_th_* = Energy Threshold3:Let *R_a_* = Active Route4:Let *R_b_* = Alternative Route5:**if**
*R_a_*.energy = *R_b_*.energy **then**6: **if**
*R_a_*.hop Count > *R_b_*.hop Count + *HCdiff_max allow_***then**7:  **if**
*R_a_*.weakLinks ≥ *R_b_*.weakLinks **then**8:   Shift To Route(*R_b_*)9:  **end if**10: **end if**11:**else if**
*R_a_*.energy < *R_b_*.energy **then**12: **if**
*R_a_*.hop Count + *HCdiff_max allow_* ≥ *R_b_*.hop Count **then**13:  **if**
*R_a_*.weakLinks ≥ *R_b_*.weakLinks **then**14:   Shift To Route(*R_b_*)15:  **end if**16: **end if**17:**else if**
*R_a_*.energy > *R_b_*.energy and *R_a_*.energy ≤ *R_b_*.energy + *E_th_***then**18: **if**
*R_a_*.weakLinks ≥ *R_b_*.weakLinks and *R_a_*.hopCount > *R_b_*.hopCount + *HCdiff_max allow_***then**19:  ShiftToRoute(*R_b_*)20: **end if**21:**end if**


## 4. Performance Evaluation

REL is evaluated through a testbed (small-scale) and OMNET++ simulator (large-scale). The testbed results are used to calibrate the simulation experiments, and also to analyze the performance of REL in real experiments. The objective of the simulation experiments is to evaluate the REL and compare it with both AODV and LABILE in terms of energy-efficiency, latency and data delivery in a large-scale scenario.

### 4.1. Parameter Calibration

Testbed experiments were conducted to find *LQI_th_* and *E_th_*, since they can be used to evaluate the link quality in real-world deployments in the presence of interference and moving people, as expected for IoT scenarios. Additionally, in testbed experiments, the energy consumption can be calculated with real hardware used by many networks.

**Figure 2.**
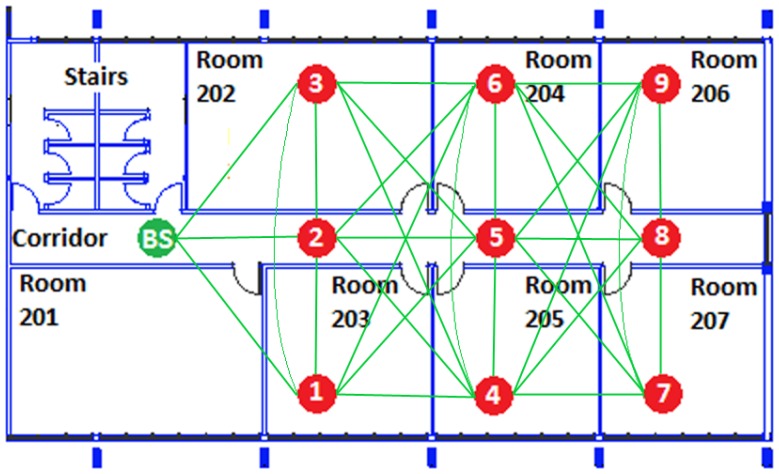
Testbed topology and connectivity map.

The experiments were carried out in the 2nd floor of the Computer Engineering Department at the Federal University of Pará. The experiments consisted of 10 SunSPOT motes [31] in a grid topology of3 × 3 and the BS on the edge, as depicted in Figure 2. The proposed grid topology is able to provide a minimum number of 5 neighboring nodes for each node.

**Figure 3.**
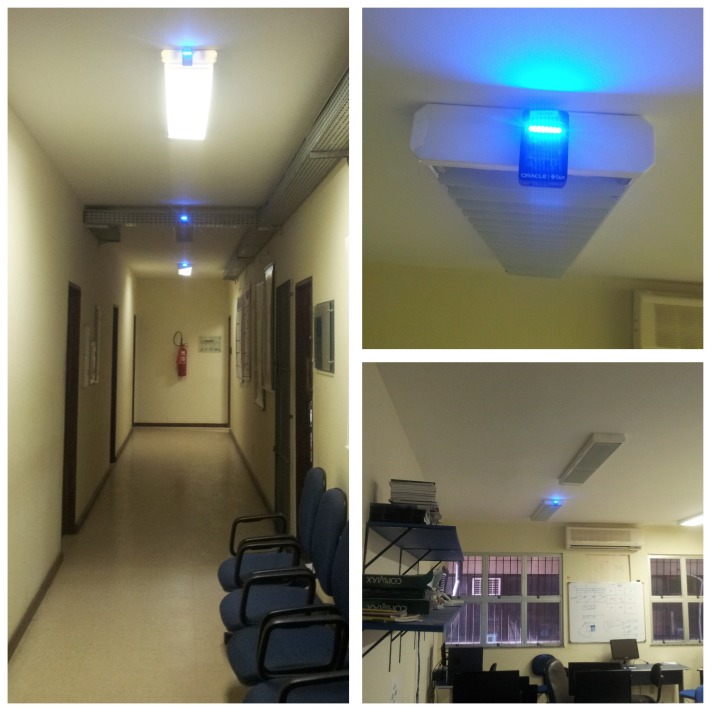
Testbed scenario on Computer Engineering Department

The experiments were carried out in an indoor environment, as shown in Figure 3. In this scenario, the nodes were deployed on the roof of the rooms and in the corridor. The experiments were conducted in a real world environment in the presence of continuous movement of people and other wireless network devices, which caused noise and interference.

**Figure 4.**
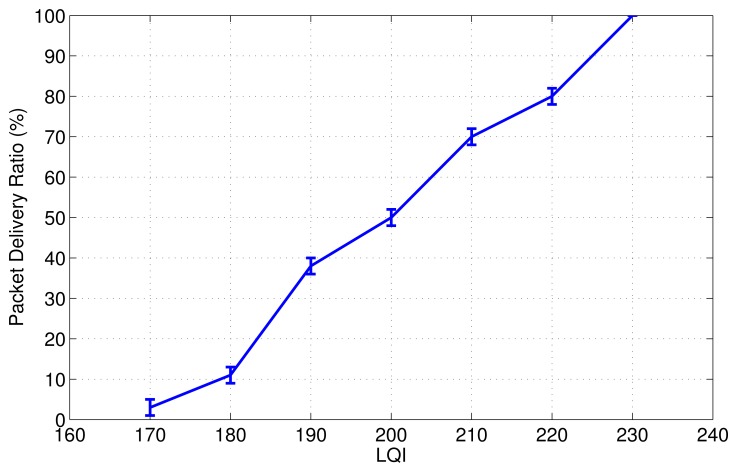
Testbed experiment to find *LQI_th_*.

As mentioned earlier, most of the IoT applications require reliable communications systems to allow the end-user to analyze the accurate collected data by the sensor nodes. We assume that most of the IoT applications need a PDR that is higher than 80% as QoS requirements (this value can be easily changed for different applications). In this context, experiments were conducted to find the LQI threshold for this purpose. Based on the results shown in Figure 4, we selected *LQI_th_*= 220.

**Figure 5.**
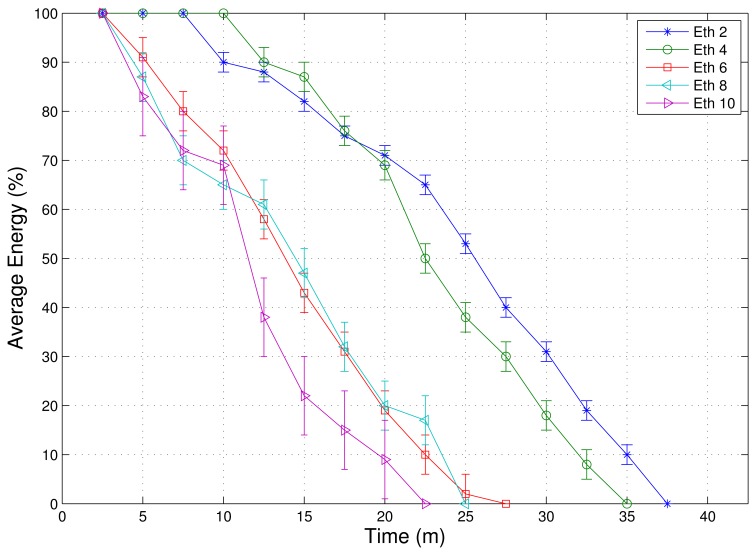
Testbed experiment to find *E_th_*.

Experiments were also conducted to find the *E_th_* in the real hardware since it is possible to analyze energy consumption in a real device. Figure 5 shows five possible values for the energy threshold varying from 2 to 10. Values of *E_th_* higher than 6 reduce the network lifetime. This occurs because of the low frequency of the RADV notifications, which provides an unbalanced consumption of network resources,degrades the performance of the load balancing mechanism, and the use of multiple routes. As result,nodes located at potential energy holes take a longer time to be detected. On the other hand, *E_th_*= 2 has the highest lifetime of all, exceeding the value 4 by 2 min.

### 4.2. Testbed Experiments

Testbed experiments were also conducted to evaluate REL and compare it with AODV in terms of network lifetime and data delivery in experiments into real environment, as expected for IoT and ubiquitous computing applications. The experiments were set up to allow each node to send 1200 packets to the BS with an inter packet interval of 0.5 s. This configuration is common for most IoT applications that require a high data rate.

**Figure 6.**
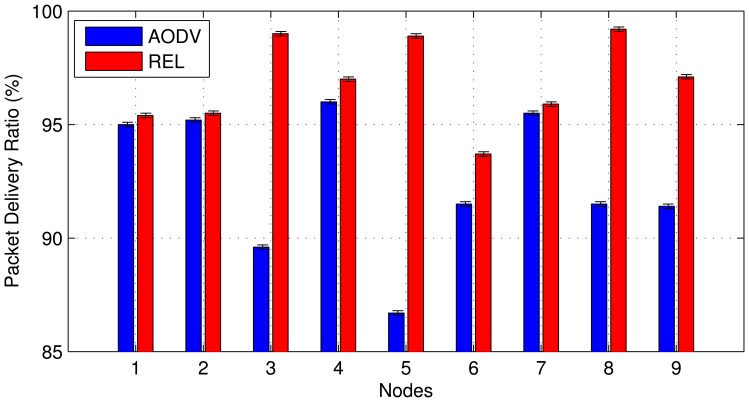
Packet delivery ratio in testbed experiments.

Figure 6 shows the results of PDR for each node when they use REL or AODV as the routing protocol. It important to point out that although PDR ranges from 0% to 100%, Figure 6 establishes an interval from 90% to 100% to make the performance evaluation clearer. On the basis of the results of Figure 6,the total packet loss of AODV is 67.6% and REL is 28.3%. Thus, REL increases the PDR by 39.3%. This is due to the fact that REL selects routes on the basis of cross-layer information, with end-to-endlink quality estimation (QoS-aware), unlike AODV that only chooses paths based on hop count. Thus,REL is able to provide data transmission with a higher degree of reliability in IoT scenarios.

The PDR of node 5 has a higher value (13.3%) of packet loss using AODV as the routing protocol,and only 1.1% using REL. As depicted Figure 2, node 5 is the central node of the grid topology used forth experiments. REL selects routes together with load balance operation, and, thus avoids continually sending packets to the same node, which causes interference, collisions, and buffer overflow. On the other hand, AODV selects routes that are only based on the minimum number of hops, therefore nodes7, 8 and 9 often send packets to node 5.

**Figure 7.**
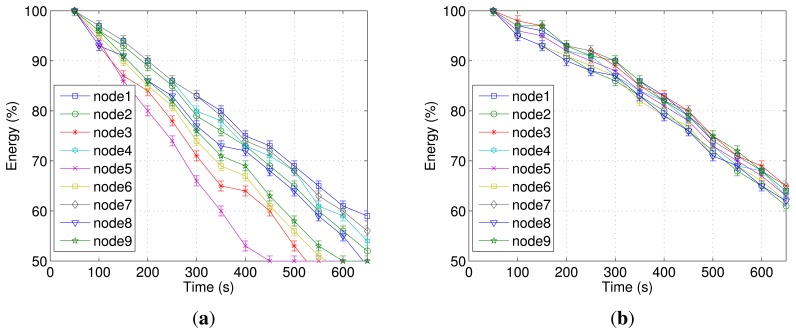
Network lifetime in testbed experiments, (**a**) AODV, (**b**) REL.

The Figure 7 shows the energy-efficiency for this testbed. AODV presents unbalanced energy consumption between nodes and their possible premature death, as can be seen in Figure 7(a). Additionally, five nodes consumed up to 50% of the energy resources before the end of the experiment. As expected, these nodes are the same as those with the highest packet loss values. Thus, routes less reliable and more susceptible to errors require more retransmissions and spend more energy.

In contrast, REL increases the network lifetime by more than 15% compared with AODV and also presents an almost uniform distribution of energy consumption, as shown in Figure 7(b). This is due to the fact that REL has a mechanism for opportunistic piggybacking, which leads to an overhead reduction and, thus, improves the energy consumption. Additionally, REL uses a load balancing scheme that provided uniform energy consumption for all network nodes.

Finally, the difference between the higher and lower energy consumption for REL was 6% (nodes 1 and 2), whereas for AODV this value exceeded 23% (nodes 3, 4, 6, 8, and 9). This is due to the reasons explained above.

### 4.3. Simulation

Simulation experiments were conducted to analyze the performance of REL using the Castalia Framework [32], which is a widely used network simulator for WSNs based on OMNET++simulator [33]. The simulations were carried out and repeated 20 times with different random seed numbers in order to obtain a confidence interval of 95%.

Due to limited available nodes in the testbed, simulation experiments were conducted to evaluate the REL and compare its performance with AODV and LABILE in terms of energy-efficiency, latency and data delivery in a large-scale scenario, *i.e.*, using a monitoring area of 100 m × 100 m and upto 200 nodes. AODV is a standard reference, where can be considered as a benchmark solution for flat routing protocols and scenarios. The LABILE protocol presents an assessment strategy based onend-to-end link quality, thus making it suitable and essential for assessing and comparing its performance with REL.

The values for energy and LQI thresholds in the simulations were collected in the testbed experiments. Table 1 shows the threshold values and other simulation parameters. The topology was generated by using Bonn Motion [34] tool, where nodes are uniformly distributed in the simulation.

Similar to LQI and energy thresholds, REL has also a hop count threshold, which cannot be found in the testbed experiments due to of the limited number of nodes. Thus, simulation experiments were performed to find *HCdiff_max___allow_*, which is able to provide a reasonable number of alternate routes without affecting the latency and PDR.

**Figure 8.**
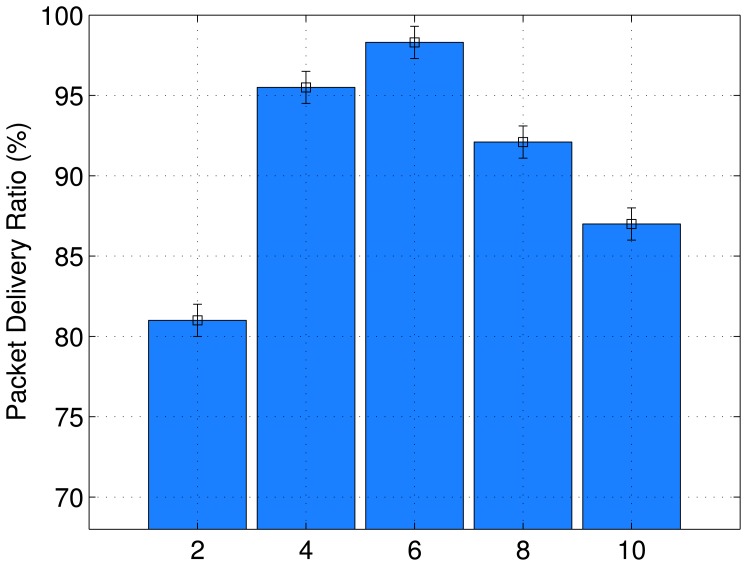
Simulation results of *HCdiff_max___allow_*.

Figure 8 shows PDR for different values of *HCdiff_max___allow_*, which ranges from 2 to 10 hops. All possible values have a PDR higher than desired, *i.e.*, 80%. There is a need to choose a suitable value for *LQI_th_*, which creates a large number of possible routes to be used for the load balancing scheme. However, values higher than 6 hops create more routes and the signaling overhead can have an effect on the network lifetime and latency. On the basis of our results, the most suitable values for *HCdiff_max___allow_*are 2 and 4 hops. In the case of our simulation experiments, we chose *HCdiff_max___allow_* of 4 hops to avoid a small number of alternate routes. The value 4 is able to consider more routes and achieve a better PDRresult, as shown in Figure 8.

Starting with the scenario of Table 1, other configurations were setup to assess the performance of REL compared with AODV and LABILE. The additional scenarios included configurations that changed the number of nodes to 20, 40, 60, 80, and 200. The purpose of this variation was to have scenarios with different node traffic demands as expected in real IoT systems. During our data analysis, the scenario with 20 nodes was used to check the accuracy of the simulation results, by comparing them with the testbed experiments.

Additionally, the scenarios that have a different number of nodes create alternate routes that are useful to evaluate the load balancing and energy-efficiency mechanisms. The performance of REL was compared with AODV and LABILE in terms of network lifetime, latency and PDR.

The WSNs/IoT applications have typically heterogeneous traffic characteristics, which can be file transfer or sensing data. Given this fact, protocols developed for these scenarios should regard the latency as an important metric. In this context, during our experiments, we evaluated the latency of REL, AODV and LABILE. Each received packet was categorized into one of the three latency categories presented in Table 2. This classification aims to clarify the performance analysis of the evaluated protocols.

For scenarios with 20 nodes, results show that REL improves the latency up to 0.8%, the latency between 0 and 40 ms, and 0.4% for packets above 80 ms. LABILE obtained results similar to REL for scenarios with 20, 40 and 60 nodes, where the maximum difference between them was 1% in packets with latency between 0 and 40 ms. However, if the number of nodes increases, the difference rises to 4%for scenarios with 100 nodes.

Additionally, the performance of AODV decreases by up to 17.9% compared with REL, and 13.9%compared with LABILE, for latency between 0 and 40 ms, and for scenarios with to 100 nodes. In the case where the scenarios with few numbers of nodes, the improvement of latency result is reduced due to the availability of short routes. However, in large-scale scenarios, such as 80, 100, and 200, nodes achieve good gains when the REL protocol is used.

In a scenario with 80 nodes, REL exceeded LABILE by up 2.4% in the lower delay category and 1%in the highest delay. The performance gain increases in a scenario with 100 nodes, where the differences are 4% and 1.8%, respectively. These results show the efficiency of REL for improving the usage of network resources.

In the scenario with 200 nodes, the protocol AODV reaches more than 40 ms delay in 26.4% of transmitted packets, a result 6% lower than obtained in the scenario of 100 nodes due to the high number of overhead and interference created in the densest scenario. Regarding the protocol LABILE,the difference reaches 17.5% and 21.6% compared with REL. In the category of delays up to 40 ms, REL maintains the highest performance, exceeding in 4.1% and 20.6% LABILE and AODV, respectively.

**Figure 9.**
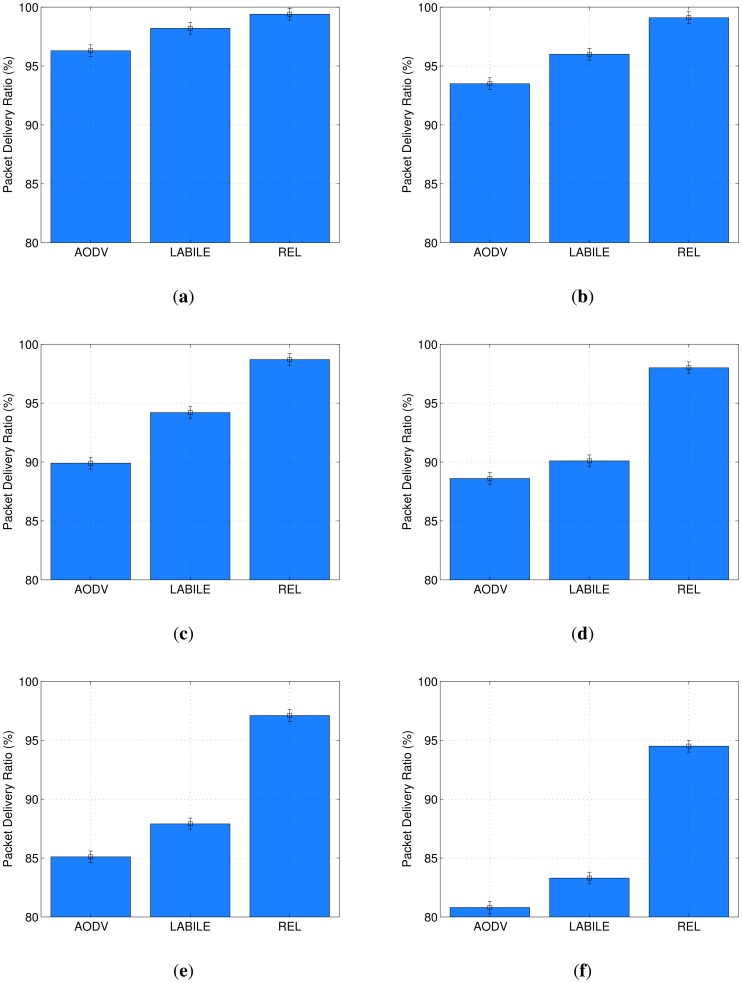
Packet delivery ratio in simulation experiments. (**a**) 20 Nodes, (**b**) 40 Nodes,(**c**) 60 Nodes, (**d**) 80 Nodes, (**e**) 100 Nodes, (**f**) 200 Nodes.

The PDR was used to measure the reliability of REL, LABILE and AODV. Figure 9 shows the PDR for all packet transmissions in scenarios with different node densities. Figure 9 establishes an interval from 80% to 100% to make it easier to analyze the results.

As can be seen in Figure 9, REL outperforms AODV and LABILE in all experiments. The difference increases when there is a rise in the number of nodes in the simulated scenario. This can be explained by the load balancing scheme and reliable route selection proposed in REL, when compared with LABILE and AODV that do not have load balancing solutions and select routes based only on link quality and the number of hops.

The scenario with 20 nodes confirms the accuracy of the simulation results in comparison with the testbed is presented in Figure 2. In the case of the testbed experiments, the average value of PDR for AODV was 92.4% and for REL was 96.8%. These results reflect the close approximation of the real and simulated experiments.

In a similar way to what was found in the latency results, there is an improvement of REL, in terms of PDR, compared with AODV and LABILE when the node density increases. These scenarios with high node density are expected for many IoT applications, such as automation of homes and offices, smart parking, healthcare and environmental monitoring.

The scenarios with 60 and 80 nodes show results that correspond to the increasing number of nodes. REL improves by 8.8% when compared with AODV and 4.5% with LABILE in a scenario with 60 nodes. In the scenario with 100 nodes, REL increases the number of packets delivered in 9.4% and 7.9%,respectively. This is due to the fact that increasing the node density also increases the number of available routes for load balancing and causes the phenomenon of signal attenuation. In this context, the lack of a technique to smooth the variation of LQI values leads to a greater switching of routes in LABILE,and thus reduces the data delivery. For these reasons, although LABILE has a mechanism to evaluateend-to-end link quality, its performance was below of the REL protocol. On the other hand, AODV selects routes that are only based on minimum hop count, which reduces its overall reliability.

The analysis showed that results with 100 nodes, *i.e.*, those with high density, had a PDR difference that reached 12% when compared with AODV, and 9.2% when compared with LABILE. Thus, if the results of the high PDR and low latency provided by REL are combined, we can observe high degrees of efficiency and reliability in its route selection and load balancing scheme. These results confirm that REL achieves a level of QoS that is appropriate for ubiquitous computing and IoT applications.

The values of the PDR for systems with 200 nodes show that REL keeps its superior performance even in dense topologies, where the PDR is around 94.5%. For the same scenarios, AODV and LABILE results were 80.8% and 83.3%, respectively.

As mentioned earlier, despite recent technological advances in embedded systems, the energy constraint remains a critical factor in WSNs/IoT, given the fact that sensor nodes have limited energy resources. Furthermore, an external energy supply is usually unavailable and the replacement of batteries is not feasible for large-scale WSNs. In this context, it is essential to have an integrated routing and load balancing scheme that can extend the network lifetime without affecting its reliability.

**Figure 10.**
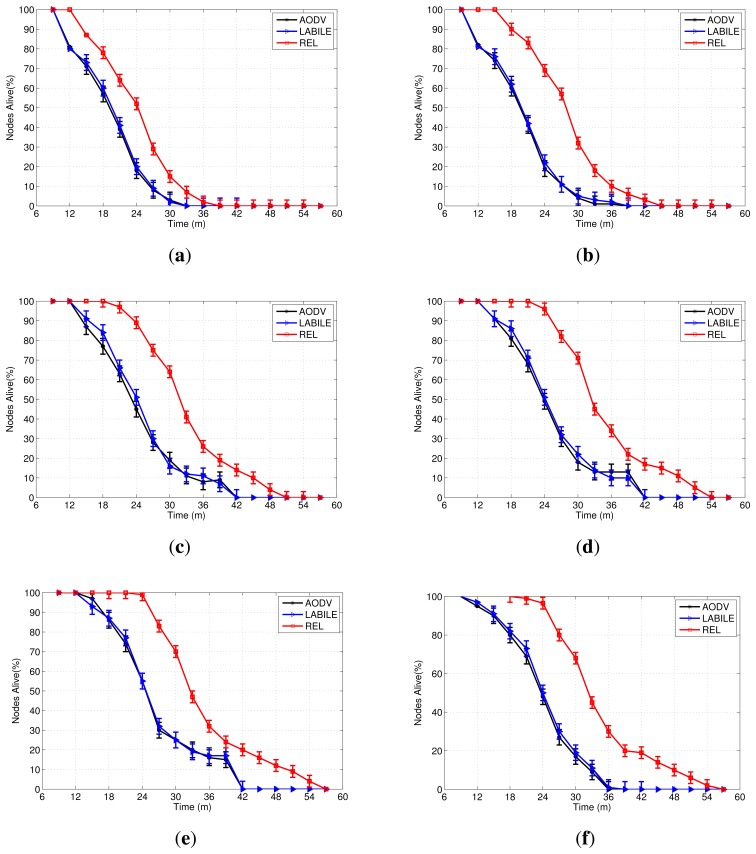
Network lifetime in simulation experiments. (**a**) 20 Nodes, (**b**) 40 Nodes,(**c**) 60 Nodes, (**d**) 80 Nodes, (**e**) 100 Nodes, (**f**) 200 Nodes.

Figure 10 shows the percentage of nodes that remained alive during the simulation. Two metrics were employed to evaluate the energy-efficiency: the entire lifetime of the network and the saturation time. The network lifetime was measured as the time spent by sensor nodes until the point when only 1% of the network nodes remains alive. The saturation time is similar to the network lifetime, although it starts from the moment of the first node death. The saturation time is useful in assessing the capacity of the routing protocols to dynamically adapt to new topologies resulting from the death of the nodes.

The lack of energy management in existing protocols, such as AODV and LABILE, means that their results can be similar in most of the experiments. The experiments outlined in Figure 10 show differences between the percentage of nodes that were alive during the saturation period for these protocols, although the difference in the network lifetime was a maximum of 1 minute. The reason for this is that LABILE is a proposal that extends the AODV protocol, but does not improved energy-efficiency features.

AODV and LABILE do not provide mechanisms that can take advantage of the large number of network nodes with respect to energy. As a result, in the scenario with 60, 80 and 100 nodes(Figure 10(c–e)) these protocols do not exceed a lifetime of 42 min. Furthermore, the saturation time also remains stable.

When the protocol REL is used, the saturation time in these three scenarios with more nodes also increased by up to 8 min. The saturation time reached 32 min for scenarios of 60 and 80 nodes, while the scenario with 100 nodes reached a maximum of 36 min.

Since the beginning of the saturation period occurs at the moment of the death of the first node, it is also included in the assessment of the energy-efficiency of a protocol. The maximum time spent until the first death, when the protocols AODV and LABILE were used, was 12 min. For the REL protocol, the minimum value was 18 min in the scenario with 20 nodes, and 22 min in topologies with more nodes.

The beginning of saturation occurs earlier in the scenario with 200 nodes compared with the scenario with 100. The instant 18 starts to decrease the number of nodes alive and extends up to the instant56. The first death occurs firstly in the scenario with 200 nodes due to interference generated by the large number of nodes. However, the saturation time is high due to the large number of alternate routes,a feature that favors the load balancing and adaptability of the REL. Protocols AODV and LABILE showed a reduction in total network lifetime, decreasing by up to 8 min.

## 5. Conclusion and Future Works

This article has presented a routing protocol together with load balance scheme based on energy and link quality (REL) for IoT applications, such as automation of comfortable homes and offices, healthcare,environmental monitoring, and smart parking. REL combines a reliable scheme for route discovery and load balance mechanism, which provides high reliability, QoS-awareness and energy-efficiency Moreover, it proposes an end-to-end route selection scheme based on cross-layer information with a minimal overhead. Nodes become energy efficient by sending the residual energy to their neighboring nodes with the aid of a piggyback and on-demand scheme. Additionally, REL also uses an event-drivenmechanism to provide load balancing as a way to improve the system performance and avoid the energy hole problem.

REL was evaluated by using simulation and testbed experiments to show its effects and benefits when compared with existing solutions. Testbed results were used to calibrate and confirm the accuracy of the simulation experiments, as well as present the impact of REL in real scenarios. At the sametime, simulation experiments were useful to evaluate REL and compare it with well-known AODV and LABILE protocols, in terms of energy-efficiency, latency and data delivery in large-scale scenarios as expected for many IoT applications. In large-scale networks with a high node density, the results showed that REL increases the network lifetime by up to 26.6%, latency by 17.9% and packet delivery by 12%,compared with AODV and LABILE.

As future studies, we will implement and evaluate REL in a large-scale test-bed, such as Wise bed and Smart Santander. We will also manage the radios/transmissions to improve the energy saving process.

## Figures and Tables

**Table 1.** Simulation parameters.

**Parameter**	**Value**
Simulation Area	100 m × 100 m
Total Number of Nodes	100
Simualtion Time	60 min
Basestation Location	(50, 50)
Topology	Uniform
Inter Packet Interval	2s
Initial Energy	18,729 J (2 AA batteries)
*E_th_*	2
*LQI_th_*	220
*HCdiff_max_allow_*	4
Radio Model	CC2420

**Table 2.** Latency.

Protocol	**Number**	Latency		
	of Nodes	0–40 ms	40–80 ms	>80 ms
	20	99%	0.6%	0.4%
	40	94.5%	4.3%	1.2%
AODV	60	90.6%	5.4%	4%
	80	85.4%	8.2%	7.4%
	100	80.6%	10.5%	9.9%
	200	74.6%	14.1%	12.3%

	20	99.5%	0.5%	0%
	40	98.7%	0.9%	0.1%
LABILE	60	98%	1.1%	0.9%
	80	96.3%	2.5%	1.2%
	100	94.5%	3.5%	2%
	200	91.1%	5%	3.9%

	20	99.8%	0.2%	0%
	40	99.4%	0.3%	0.1%
REL	60	99%	0.6%	0.4%
	80	98.7%	1.1%	0.2%
	100	98.5%	1.3%	0.2%
	200	95.2%	3%	1.8%
